# Patients’ anticipated actions following transient ischaemic attack symptoms: a qualitative vignette-based study

**DOI:** 10.1186/s12875-017-0594-4

**Published:** 2017-02-03

**Authors:** Parker Magin, Terry Joyce, Christopher Levi, Daniel Lasserson

**Affiliations:** 1 Discipline of General Practice, University of Newcastle, University Drive, Callaghan, 2308 NSW Australia; 2 Centre for Translational Neuroscience and Mental Health, University of Newcastle, University Drive, Callaghan, 2308 NSW Australia; 3 Nuffield Department of Medicine, University of Oxford, Oxford, Oxon UK

**Keywords:** Family practice, General practice, Ischaemic attack, transient, Stroke, Delivery of health care, Health care quality, Access and evaluation, Health behaviour

## Abstract

**Background:**

Transient Ischaemic Attack (TIA) requires urgent investigation and management. Urgent management reduces the risk of subsequent stroke markedly, but non-presentation or delays in patient presentation to health services have been found to compromise timely management. We aimed to explore general practice patients’ anticipated responses to TIA symptoms.

**Methods:**

This was a qualitative study employing semi-structured telephone interviews. Participants were recruited from respondents in an earlier quantitative study based in Australian general practices. Maximum variation purposive sampling of patients from that study (on the basis of age, rurality, gender and previous experience of stroke/TIA) continued until thematic saturation was achieved. After initial interviews explored knowledge of TIA and potential responses, subsequent interviews further explored anticipated responses via clinical vignettes containing TIA and non-TIA symptoms. Transcribed interviews were coded independently by two researchers. Data collection and analysis were concurrent and cumulative, using a process of iterative thematic analysis and constant comparison. A schema explaining participants’ anticipated actions emerged during this process and was iteratively tested in later interviews.

**Results:**

Thirty-seven interviews were conducted and a ‘spectrum of action’, from watchful waiting (only responding if symptoms recurred) to summoning an ambulance immediately, was established. Intermediate actions upon the spectrum were: intending to mention the episode to a general practitioner (GP) at a routine appointment; consulting a GP non-urgently; consulting a general practitioner (GP) urgently; and attending an Emergency Department urgently. The substrate for decision-making relating to this spectrum operated via three constructs: the ‘individual set’ of the participant (their inherent disposition towards action in response to health matters in general), their ‘discriminatory power’ (the ability to discriminate TIA symptoms from non-TIA symptoms) and their ‘effective access’ to health-care services.

**Conclusions:**

Policies to improve patients’ accessing care (and accessing care urgently) post-TIA should address these three determinants of anticipated action.

**Electronic supplementary material:**

The online version of this article (doi:10.1186/s12875-017-0594-4) contains supplementary material, which is available to authorized users.

## Background

Transient ischemic attack (TIA) can confer a high risk of stroke immediately post-event with an average 11·0% risk during the first 7 days [1]. Furthermore, the outcomes after stroke preceded by TIA have been reported to be worse than in those without prior TIA [2]. Urgent preventative treatment post-TIA can confer marked risk reduction, [3–5] therefore current Australian guidelines [6] recommend urgent investigation and initial treatment of TIA, ideally within 24 h of the initial event.

Assessment delay post-TIA is more often due to patient delay than to health services delay [7]. Following a TIA in a United Kingdom (UK) study including a community-based sample, 44.4% of people delayed seeking medical attention for 24 h or more [8]. In a wholly community-based (UK) study, [9] 33% delayed presentation for greater than 24 h. The majority of patients with TIA present to GPs rather than Emergency Departments (EDs) [9]. Patients particularly delay presentation when attending general practice rather than EDs [10] and, if presenting to general practice, at night and on weekends [8, 11]. ED presentations are also delayed on weekends [10]. Motor symptoms, longer duration of symptoms, and higher risk TIAs are associated with less delay [8–10] as are purely visual symptoms, [10] but correct recognition of symptoms has been inconsistently associated with delay [8, 9].

These previous studies of TIA (and similar studies of stroke [12, 13]) have examined patient behaviours and delays in patients’ presentation following TIA, but have studied only patients who *did* present to medical care. There is evidence from population-based studies that a majority of patients with symptoms suggestive of possible TIA do not present to medical care [14, 15]. Thus we chose to explore anticipated actions in an unselected population rather than one accessing care following an actual TIA.

The aims of this study were to explore *anticipated* actions in response to TIA symptoms, and the context of these actions, in adults attending Australian general practices.

## Methods

This was a descriptive qualitative study [16] employing individual semi-structured interviews, conducted by telephone.

### Recruitment

Participants were recruited from participants in an earlier questionnaire-based cross-sectional quantitative study [17]. That study had recruited consecutive adult attendees (*n* = 869) at 14 of the 16 member practices of the Hunter New England Central Coast Network of Research General Practices, [18] covering urban/rural areas and diverse socioeconomic demographics in a region of New South Wales, Australia. Participants in the questionnaire study who expressed interest in the interview were selected for participation via purposive maximum variation sampling [19] based on demographic data (age, rurality, gender and previous personal or family experience of stroke/TIA).

### Data collection

Telephone interviews were conducted by a single researcher (TJ). The interview structure included discussion of access to, and determinants of use of, health services generally. In an initial exploratory series of twelve interviews knowledge of, and experience of, stroke and TIA symptoms and anticipated actions in the event of these symptoms were explored. The majority of these twelve participants had personal or family experience of stroke. In subsequent interviews (with further participants, most of whom had no personal or family history of stroke), discussion was prompted by participants being presented with a number of clinical vignettes (involving themselves or a friend they were with at the time of the symptoms), asking the participant to describe how they would respond to each. Analysis of the twelve exploratory interviews, as well as clinical considerations, informed the construction of the vignettes. Use of vignettes is valuable in qualitative studies not only in eliciting anticipated actions but in refining an understanding of people’s attitudes, perceptions, and beliefs [20].

Many of the vignettes used in this study contained TIA-consistent symptoms, but others were consistent with non-TIA events. Responses to non-TIA vignettes (which were introduced sparingly compared to TIA vignettes) provided context for TIA responses, including to what extent anticipated response to TIA symptoms were influenced by generic care-seeking as opposed to TIA-specific care-seeking factors. Previous responses to any past stroke or TIA onset (either personal or witnessed) were also elicited.

The clinical topics of the individual vignettes are presented in Table 1. There were more vignettes than could be discussed in any single interview – the rationale was to elicit data on responses to a broad spectrum of heterogeneous TIA experiences. A broad coverage of these scenarios was sought.

**Table 1:** Scenarios presented during interviews

Likely TIA scenarios (lasting 5 min) • Speech disturbance – expressive • Speech disturbance – receptive • Dense hemiplegia +/- dysphasia • Unilateral upper limb paresis • Ataxia and incoordination • Incoordination • Dressing apraxia • Other apraxia • Amaurosis fugax	
Non-stroke scenarios • Acute coronary syndrome (crushing chest pain for 20 min) • Simple vaso-vagal faint • Classical migraine with fortification spectra • GI upset with diarrhoea and vomiting • Respiratory tract infection/possible pneumonia • Dysphagia without other neurological symptoms	

However, the number of scenarios, the particular scenarios, and the order in which they were presented in any individual interview were not fixed, but were at the discretion of the interviewer who could utilise any individual clinical scenario to explicitly explore issues arising in the participant’s responses to previous scenarios (discussions were informant-led as far as possible).

As data-collection progressed, and as new areas of interest emerged, additional themes for exploration were added to the interview schedule. There was a fundamental change in interview schedule between the initial exploratory interviews and the later vignette-based interviews, but the schedules for both initial exploratory and vignette-based interviews developed iteratively from interview to interview. The vignettes available for use in the interviews remained the same throughout the vignette-based phase of data collection.

Interviews were recorded to audiotape and transcribed verbatim. Recruitment continued until thematic saturation had been achieved.

### Data analysis

Thematic data analysis [21] was cumulative and concurrent throughout the data collection period using an inductive approach [21]. After a process of reading and re-reading of the transcripts [16, 21, 22] we performed a line-by-line analysis of meaning of individual pieces of data and collated these units of meaning. From this collation, we derived a set of first order codes which we applied to the transcripts [21]. This involved development and application of a code-book and use of a process of constant comparison [22]. Constant comparison involved comparing the emerging codes between each transcript. An iterative modification of the code book continued throughout this process. Analysis then involved an examination of relationships and interactions between codes and the construction of an explanatory schema [21] which was tested against further interview data, [21, 22] with subsequent iterative modification of the schema.

Each transcript was separately coded [23] by two members of the research team (TJ and PM). Differences in researcher perspective were resolved by mutual agreement [24]. Reflexivity [25] was inherent in this process via awareness and consideration of the influence of the researchers’ respective professional and academic backgrounds (PM a GP, and TJ a nurse). The different clinical backgrounds of the researchers brought different clinical perspectives to the interpretation of participants’ responses.

## Results

Thirty-seven interviews were conducted. The demographics of the participants are presented in Additional file 1: Table S1.

**Fig. 1:**
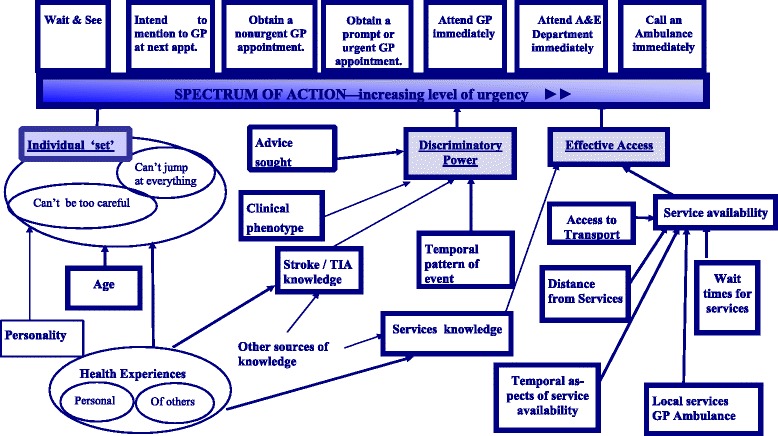
The spectrum of anticipated action in response to TIA symptoms

The explanatory schema, constructed from the interview data, of participants’ anticipated actions in the event of TIA symptoms is presented in Fig. 1.

### Spectrum of anticipated action

The overarching finding was of a ‘spectrum of anticipated action’ in the event of TIA symptoms. The spectrum extended from no action and watchful waiting (‘Wait and See’) through non-urgent and urgent accessing of GP care to urgent ED attendance (at the most urgent end of the spectrum, via ambulance).

Calling an ambulance was a quite common anticipated response to TIA scenarios, though some participants would elect private transport to the ED.

When things like this happen [amaurosis fugax], you don’t muck about and wonder where you’re going to go. You ring an ambulance automatically. (Interview 26: male, age 80s)

in that case [a TIA with ataxia and incoordination] I’d sort of suspect a stroke. And probably whisk them off to hospital…..probably in my car.[…] I’m not sure if it’s worth calling an ambulance for. (Int 4: female age 60s)

Others felt it was appropriate to be seen by a GP, either immediately, or non-urgently, or opportunistically next time they had a GP appointment.

I’d probably put them [a friend with a TIA with ataxia and incoordination] in the car and take them down to the local GP…… I’d probably still get them down to the medical practitioner to get them checked over as quickly as possible. (Int 2: male age 60s)

So it [amaurosis fugax] resolved …I think I’d still try to make an appointment and get it checked out within a couple of days. (Int 12: female: age 70s)

I would certainly mention it [Dressing apraxia] to the doctor next time I saw her. (Int 16: female: age 70s)

Some participants would defer action (‘wait and see’) to some TIA scenarios, especially in the absence of further symptoms.

I’d be concerned but I’d just monitor it [amaurosis fugax] and just see whether it was just a one off thing that was happening and if it recurred or if it was sustained then I’d need to contact either my GP or the eye fellow. (Int 14: male, age 60s)

The substrate for decision-making relating to this spectrum was complex, but can be seen to operate via three constructs: the ‘individual set’ of the participant (their inherent disposition towards action in response to health matters in general), their ‘discriminatory power’ (the ability to discriminate TIA symptoms from non-TIA symptoms) and their ‘effective access’ to health-care services.

### Individual set

The ‘individual set’ denotes the individual’s propensity to choose actions at a particular point, or in a particular range, of the spectrum of action. This was characterised as a continuum from those with the lowest threshold for action (you ‘can’t be too careful’), to those with a more graduated, and pragmatic, set of responses (‘you can’t jump at everything’).

in circumstances like this the emphasis would be on making decisions as quickly as possible and I’d rather make a wrong decision than be sorry. I’d rather make a decision in haste, erring on the side of caution than not acting and regretting it later. (Int 7: male, age 60s)

you can’t just jump every time you see some symptom that vaguely might be connected perhaps with a stroke. (Int 3: female, age 50s)

The ‘can’t be too careful’ set became particularly evident if the participant was making decisions about responding to the symptoms of others rather than themselves.

I wouldn’t be prepared of having the responsibility of it, you know? (Int 8: female, age 50s)

Factors contributing to the individual set included past health experiences (either personal or observed in family and friends) that involved experiences of good outcomes for serious illness promptly assessed and treated or, conversely, experience of having ‘panicked’ over symptoms that proved non-serious. These experiences may not have related to neurological illness or symptoms.

And the ambulance guy said I did the right thing. The only thing to do is immediately to ring the ambulance [an episode of pulmonary embolism]. (Int 1: male, age 40s)

I had flashes in my left eye and I saw my opthalmist and he told me it was this, that and the other. Nothing to be concerned about. It was just wear and tear on my body. (Int 14: male, age 60s)

Health experience via perceptions of personal risk also contributed to personal set.

I’ve got type 1 diabetes so I need to know what I should be doing if and when I get the stroke or heart attack. (Int 4: female, age 60s)

The most prominent determinant of perceived risk was increasing age.

as we’re getting older we become more and more aware of the potential for things going wrong, as you’re mixing more and more with people that are older and older. (Int 7: male, age 60s)

But the most important determinant of individual set was the participant’s inherent predisposition to respond to health issues in general. Some participants’ responses displayed considerable stoicism and their anticipated actions tended towards the ‘can’t jump at everything’ end of the spectrum.

I gradually came good. Just by sheer will power. That’s the problem with me. I just accept things and I get on with it and yeah, I’ll deal with it later sort of thing. (Int 5; female, age 70s, describing her response to an actual aphasic TIA)

### Discriminatory power

Anticipated response to TIA symptoms along the spectrum action was dependent upon an assessment of the nature of the episode, if not a specific diagnosis then an assessment of need for medical attention and the urgency of that attention. Participants generally found this task quite challenging.

you don’t really know do you? So you need to, you need to address it. (Int 10: female, age 60s)

It’s just knowing [or] trying to work out the difference between what needs immediate medical intervention right now and what needs intervention. (Int 3: female, age 50s)

Ability to discriminate TIA/stroke from non-vascular events and urgent from non-urgent events was modest in this study. Parameters that underpinned participant’s anticipated actions were the clinical phenotype of the event, the temporal pattern of event (or events), the participant’s medical/stroke-specific knowledge levels, which in some cases would be supplemented by the seeking of advice.

For some clinical phenotypes, there was clear recognition of both the seriousness of the symptoms and the need for immediate hospital access (usually by ambulance) and the presumptive diagnosis. This was universally so for the hemiplegia and ischaemic-character chest pain scenarios.

That’s a heart attack. I’d call an ambulance. (Int 6: female, age 70s)

[Weakness] on one side, definitely ambulance. (Int 15: female, age 50s)

For other scenarios, recognition of the likely diagnosis and/or appropriate action was less clear-cut. A faint or migraine with fortification spectra was as likely as most TIA scenarios to prompt the calling of an ambulance.

[I’d think]some sort of massive infection or, again, I had some sort of terrible haemorrhage or something like that.I’d be…I would be really worried about it [migraine with fortification spectra]. (Int 11: female, age 70s)

Within TIA scenarios, hemiplegic symptoms (as above) prompted immediate recourse to ED via ambulance. At the other end of the spectrum, more subtle symptoms were assessed as requiring less urgency – in fact, less urgent than some non-TIA symptoms such as syncope.

It [alexia] could be a number of things and I’d monitor it, keep an eye on it. (Int 14: male, age 60s)

Probably think nothing of it [dressing dyspraxia]. [I wouldn’t seek help] just for buttons (Int 22: male, age 60s)

For the acute neurological symptoms and chest pain, participants were initially asked about their anticipated responses without specifying a time frame. They were then asked about their responses should the symptoms spontaneously resolve. For some participants, the temporal pattern affected their response. This could be ‘watch and wait’, but was usually a down-grading along the spectrum of action (for example, organising a GP appointment rather than calling an ambulance).

If [amaurosis fugax] lasted longer than half an hour I probably would have gone to the polyclinic. (Int 31: female age 40s)

Well, after I got better [resolution of expressive dyspahasia] I’d contact and make an appointment with my GP as soon as possible. (Int 14: male, age 60s)

The downgrading effect of resolution of symptoms applied even for ‘crescendo’ (recurrent) TIAs.

I would consider that [an episode of dressing apraxia followed later in the day by an episode of dyspraxia] serious enough to see a GP [but not a more urgent response]. (Int 16: female, age 70s)

But for many participants a scenario with resolution of symptoms did not change their decision-making, even if that involved calling an ambulance. For a TIA involving hemiplegia or ischaemic-character chest pain all participants would call an ambulance. Even in other scenarios, resolution of symptoms didn’t necessarily alter anticipated responses.

I’d still suggest that they get to the hospital really. [for a TIA with ataxia and in-coordination]. (Int 8: female, age 50s)

There was a suggestion that a ‘can’t be too careful’ individual set operated in modulating the effect of this temporality.

So you know, things can happen. I guess people can be a bit blasé about their own health. Particularly if they start feeling better in a few minutes and want to ignore it. Don’t they? Human nature. (Int 21: female, age 50s)

The level of medical or stroke-specific knowledge was generally modest (and mainly related to personal or family history) and thus not a prominent influence on spectrum of action. A few respondents could recall having seen or heard the public education ‘FAST’ (Face, Arm, Speech, Time) campaign, and may have recalled that an ambulance should be called, but only one could recall ‘face, arm, speech’ as the symptom elements of the acronym.

A few participants would ask for advice (usually friends and neighbours), but very few would phone a GP practice for advice on what action to take.

I’d be looking for advice [from the GP] over the phone and then they’d tell me if I needed an urgent appointment. (Int 21: female, age 50s)

### Effective access

For many participants their anticipated responses were influenced by pragmatic decisions based on effective access to services. This, in turn, was determined by knowledge of services as well as availability. Availability was affected not only by the existence of services locally but by their hours of operation and waiting times

I guess if the GP wasn’t there [after a TIA with dysphasia] I would have gone into the pharmacy and sat down and got them to check my blood pressure (Int 5: female, age 70s)

if you get an ambulance and you go up to the hospital you have to wait there for 8 or 9 h anyway and …and it’s full of infections so why would you rush to the hospital? (Int 5: female, age 70s)

‘Cos if you wait for the GP you might have to wait a couple of days get an appointment and you might be dead by then. (Int 1: male, age 40s) and by participants’ distance from services and access to transport.

I’m living in [rural area] and I don’t always have a neighbour nearby and we’ve only got one car. So sometimes it can be a little bit isolated. So I would ring an ambulance. (Int 11: female, age 70s)

Effective access also involved the accessed service being perceived as appropriate for TIA management.

[GPs] don’t have all the equipment if you need a scan and things like that, they refer you anyway. ‘Oh, you need a scan, need an x-ray’. (Int 9: female, age 50s)

## Discussion

### Summary

Our study established a spectrum of anticipated action following TIA that was predicated on the individual’s inherent disposition (set) to health-seeking, their ability to discriminate cerebrovascular from non-cerebrovascular symptomatology, and their effective access to appropriate health care. Apart from motor symptoms (hemiplegia), discrimination was modest and here the principal determinant of action was individual set. Many of the participants’ anticipated responses (immediate ED or GP attendance) would have been in keeping with guidelines for investigation and management within 24 h. But a considerable number of participants would have delayed presentation (leaving them vulnerable to early stroke occurrence) or not sought medical attention at all.

### Strengths and limitations of the study

The qualitative methodology was a strength. Previous quantitative studies have demonstrated that delay in TIA presentation is a considerable problem and established associations of delay, but are unable to explore the reasoning and complexity behind patients’ behaviours. The transferability of results is likely to be good in health care systems such as Australia’s as application of the findings will be in community settings and our study details responses of participants recruited from a broad general practice setting and contextualizes participants’ responses within that setting. General practice in Australia, as in many countries, is the cornerstone of the health system and serves a gatekeeper function to secondary care. For an urgent condition such as TIA where specialist secondary care is indicated by clinical guidelines,^6^ patients may access medical care through initial contact with general practice or through emergency medicine departments. Participants in our study reported anticipated use of each of these pathways to medical care frequently.

The two-step methodological approach of the initial 12 interviews eliciting data of intrinsic value in exploring the research question as well as informing the construction of vignettes to be used in the subsequent 25 interviews was an efficient process (compared to conducting separate studies).

The recruitment of non-clinical participants rather than TIA- presenting patients was both a strength and a weakness. Reported anticipated actions may not necessarily translate as actions. But a significant proportion of patients with TIA do not present to care, and their reasons for non-presentation are arguably as important as the reasons for presentation delay.

### Comparison with existing literature

A previous qualitative study of presenting patients’ experiences of TIA [26] noted that some patients had initially ignored symptoms, but did not explore this further. Previous quantitative research (in those who do present), consistent with our findings, has demonstrated appreciable delay in presentation post-TIA [8–11]. Effective access was a barrier to presentation in our study, consistent with UK findings of presentation delays for TIAs occurring out-of-hours [9, 11]. Other previous findings of associations of increased time to accessing care - non-motor symptoms, [8–10] GP rather than ED presentation [10] incorrect attribution of symptoms [9] and having someone else identify the problem [27] – are also broadly consistent with our participants’ anticipated behaviours. Similarly, in a UK TIA study sub-analysis, [9] 31% of TIA/minor stroke patients who did not present until having a recurrent stroke were less likely to have motor or speech symptoms than patients who presented after initial TIA/minor stroke.

### Implications for practice, policy and research

Addressing delays in presentation and non-presentation post-TIA should consider our study’s findings of the underpinnings of those care-seeking choices.

Effecting change in individual’s inherent disposition seems unlikely. Our findings suggest that much of improved access to services needn’t involve increased services but, rather, better triage by GPs (and communication of this to patients – see below).

Better triage by GPs may involve triage protocols that explicitly include common acute TIA symptoms but also acknowledge the variety and subtlety of neurovascular presentations (noting that many of the differential diagnoses of TIA also involve a need for urgent evaluation). Triage protocols will need to be supplemented by education of staff involved in triage (receptionists as well as practice nurses) in TIA symptomatology.

Change in discriminatory capacity via increased knowledge of TIA symptomatology and its implications and recommended responses seems an attractive option. But this may not be straightforward. Public education programs promoting urgent presentation for stroke: “time is brain”, stroke is “brain attack” and “FAST” (face, arm, speech, time), [28–30] have been running in many countries in recent years but have been found to have modest effects [29, 31, 32] on stroke knowledge and appropriate anticipated action in the event of stroke. These public education programs specifically target stroke and do not contain TIA-specific advice (that, even if the symptoms resolve spontaneously, urgent medical care should still be sought). Qualitative studies of patients who have presented to hospital with acute coronary syndrome (a clinical scenario somewhat analogous to TIA) don’t report participants having considered general practice as a care-seeking option [33, 34]. This may be attributable to secondary care recruitment methods in these studies but may also reflect the success of concerted public awareness campaigns over a period of years encouraging patients with chest pain to present urgently to hospital. Incorporating TIA-specific messages into stroke campaigns, however, has the capacity to dilute and confuse messages around the primary objective: the urgency of presentation for stroke thrombolysis. A further issue is that of poor recognition (compared to motor and speech symptoms) of posterior circulation and non-dominant parietal symptoms). Incorporation of these could further dilute and confuse the relatively straightforward message around ‘FAST’.

An alternative approach would be individually-delivered structured patient education targeted at higher-risk patients (e.g. with previous stroke/TIA/vascular disease, Atrial Fibrillation, hypertension). GP practice nurses would be an ideal option for delivering this targeted education. This education could involve the symtomatology of TIA/stroke. Further aspect of this education would be assurance that potential TIAs will be triaged as emergencies and setting out of options for urgent medical care access in- and out-of-hours. Such an intervention is a suitable subject for future research.

## Conclusions

We found that anticipated responses to TIA symptoms existed on a ‘spectrum of action’, from watchful waiting to summoning an ambulance immediately. Positioning on the spectrum was subserved by three constructs: the ‘individual set’ of the participant (their inherent disposition towards action in response to health matters in general), their ‘discriminatory power’ (the ability to discriminate TIA symptoms from non-TIA symptoms) and their ‘effective access’ to health-care services.

Policies to improve patients’ accessing care (and accessing care urgently) post-TIA should address these three determinants of anticipated action.

## Additional file

Additional file 1: Table S1. Demographics of participants. (DOCX 14 kb)

## Abbreviations

ED: Emergency Department
FAST: Face, Arm, Speech, Time
GP: General Practitioner
TIA: Transient Ischaemic Attack
