# Supplementary material for: Patients’ anticipated actions following transient ischaemic attack symptoms: a qualitative vignette-based study
Source: BMC Fam Pract. 2017 Feb 3;18:14. doi: 10.1186/s12875-017-0594-4 (PMC5291945; doi:10.1186/s12875-017-0594-4)
Supplement: Additional file 1: Table S1. — Demographics of participants. (DOCX 14 kb) [file 12875_2017_594_MOESM1_ESM.docx]

**Additional file 1: Table S1: demographics of participants (interviews incorporating clinical vignettes denoted by asterisk)**

| PARTICIPANT Interview | SEX | AGE-BAND | RURALITY | PERSONAL or FAMILY EXPERIENCE of STROKE |
| --- | --- | --- | --- | --- |
| 1* | m | 40-49 | rural | Yes |
| 2* | m | 60-69 | rural | no |
| 3* | f | 50-59 | urban | no |
| 4* | f | 60-69 | rural | no |
| 5* | f | 70-79 | urban | no |
| 6* | f | 70-79 | urban | no |
| 7* | m | 60-69 | urban | no |
| 8* | f | 50-59 | urban | Yes |
| 9* | f | 50-59 | rural | Yes |
| 10* | f | 60-69 | rural | no |
| 11* | f | 70-79 | rural | no |
| 12* | f | 70-79 | rural | no |
| 13* | f | 60-69 | rural | Yes |
| 14* | m | 60-69 | rural | no |
| 15* | f | 50-59 | rural | no |
| 16* | f | 70-79 | rural | no |
| 17* | m | 60-69 | urban | no |
| 18* | f | 80-89 | rural | no |
| 19* | f | 70-79 | urban | no |
| 20* | f | 80-89 | urban | Yes |
| 21* | f | 50-59 | rural | no |
| 22* | m | 60-69 | rural | no |
| 23* | m | 70-79 | urban | no |
| 24* | f | - | urban | no |
| 25* | f | 60-69 | urban | no |
| 26 | m | 80-89 | rural | Yes |
| 27 | f | 70-79 | rural | Yes |
| 28 | m | 50-57 | urban | Yes |
| 29 | f | 60-69 | urban | no |
| 30 | f | 80-89 | urban | Yes |
| 31 | f | 40-49 | rural | Yes |
| 32 | m | 60-69 | rural | Yes |
| 33 | m | 60-69 | urban | Yes |
| 34 | f | 70-79 | rural | Yes |
| 35 | f | 60-69 | urban | no |
| 36 | f | 40-49 | urban | Yes |
| 37 | m | 60-69 | urban | no |

*Interviews including clinical vignettes
